# Visual attention and subjective preference in mascot design: evidence from eye-tracking, ratings, and fsQCA

**DOI:** 10.3389/fpsyg.2026.1748647

**Published:** 2026-07-16

**Authors:** Chen Chen, Yuxi Lin

**Affiliations:** College of Furnishings and Industrial Design, Nanjing Forestry University, Nanjing, Jiangsu, China

**Keywords:** baby schema, eye tracking, face-like perception, fsQCA, mascot design

## Abstract

**Introduction:**

Mascot design plays an important role in brand communication, yet empirical evidence on how specific visual features shape attention and preference remains limited. This study examined how mascot type, head-to-body ratio, and eye size are associated with fixation-based visual attention and self-reported preference among young adults.

**Methods:**

The study followed a three-stage design. First, Experiments 1–3 used eye-tracking to examine visual-attention patterns related to mascot type, head-to-body ratio, and eye size. Second, a questionnaire-based preference rating study examined whether these attention patterns converged with self-reported liking. Third, Experiment 4 adopted a full-factorial stimulus structure, and fsQCA was used to identify configurational pathways associated with high total fixation duration.

**Results:**

Animal mascots, the 1:2 head-to-body ratio, and large-eye conditions showed the strongest descriptive attention and preference patterns within their respective design variables. The fsQCA results identified multiple sufficient configurations associated with high total fixation duration. A compact head-to-body ratio condition, operationalized as 1:1.5 or 1:2, appeared as a shared core condition across the sufficient pathways, although it did not meet the threshold for a necessary condition.

**Discussion:**

These findings support a configurational understanding of mascot design, suggesting that visual attention and preference are shaped by both individual design features and their combinations.

## 1 Introduction

In today's increasingly digital and visually driven landscape, the emotional connection between brands and users has become a critical dimension of market competition (Thomson et al., 2005). As a key vehicle of brand identity, mascots not only serve visual recognition functions but also subtly communicate brand personality, foster community affinity, and shape user loyalty (Park et al., 2010). Understanding which design features effectively capture and sustain viewers' visual attention is therefore a foundational step toward optimizing mascot effectiveness. However, a notable paradox exists in both research and practice: although mascots are widely employed in brand communication, design decisions often rely on intuitive experience or aesthetic preferences, lacking a scientific basis grounded in cognitive mechanisms and empirical evidence on visual attention.

This issue is particularly relevant when targeting younger consumers, who are highly responsive to digital visual content and whose aesthetic preferences are shaped by rapidly changing media environments (Golossenko et al., 2020). Therefore, systematically deconstructing how specific design elements of mascots influence visual attention among young adults, from the perspectives of visual cognition and experimental psychology, carries significant theoretical and practical implications.

This study aims to examine how selected mascot design features influence fixation-based visual attention and subjective preference among young adults. Baby schema theory and research on face perception and face-like perception are used as interpretive frameworks for discussing the observed patterns.

Baby schema theory may help interpret why relatively large heads and prominent eyes are associated with visual attention and preference in some mascot designs. Lorenz first proposed the baby schema theory. He postulated that humans have an innate tendency to exhibit positive emotions and caregiving behaviors toward stimuli possessing infantile features, such as a large head, large eyes, and a round face (Lorenz, 1943). In a pivotal experiment, Glocker et al. (2009) found that infant faces exhibiting stronger baby schema features (e.g., larger head and eyes, shorter chin) were not only judged by adults as more attractive and healthier but also triggered a significantly stronger preference for adoption. Borgi et al. (2014) revealed a consistent influence of baby schema features on the perception of both human infant and animal juvenile faces. The baby schema, as an evolutionarily conserved mechanism, already functions in preschoolers, with research by Kitada and Shimizu (2025) showing it triggers cuteness perception and an attentional preference (longer eye gaze) in children aged 4–6. Spencer et al. (2024) provided a novel epigenetic perspective on the variability in adult responses to infant cuteness, showing that methylation of the oxytocin receptor gene underlies differences in both the motivation for caretaking and the brain's reactivity to baby schema features. These findings provide developmental psychological evidence for the innate and adaptive nature of “cuteness.” Kringelbach et al. posit that the influence of the baby schema extends far beyond the parent-child relationship to encompass broader domains. Prior research on baby schema suggests that infant-like visual features, including round faces, relatively large heads, enlarged eyes, and soft contours, may influence cuteness perception, gaze allocation, and preference. Related work in visual design also indicates that such features are frequently used in cartoon characters and consumer products to create approachable and emotionally engaging impressions (Yao et al., 2025; Kanwisher et al., 1997). In the present study, head-to-body ratio was used as a controllable design proxy for compact infant-like body proportion, whereas eye size was used as a visible ocular feature related to baby-schema cues. These findings provide useful background for examining head-to-body ratio and eye size as mascot design variables. Accordingly, the present study investigated how body proportion and eye-related features are associated with fixation-based visual attention and subjective preference in mascot design.

In addition to this interpretive background, research on face perception and face pareidolia provides insights into how viewers respond to face-like cues in mascot images. Kanwisher et al. revealed that the Fusiform Face Area (FFA) in the human brain, specialized for face processing, responds strongly both to real faces and to simplified, face-like configurations, suggesting a neural basis for the tendency to perceive faces in non-face stimuli—a phenomenon also referred to as face pareidolia (Tong et al., 2000). Tong et al. further clarified the FFA's core role in structural encoding and identity recognition, thereby laying the groundwork for understanding how the visual system processes face-like cues in non-human or stylized objects (Windhager et al., 2008). Windhager et al. (2008) found that consumers anthropomorphize car front-ends, attributing specific emotions and personality traits to them, which subsequently guides their aesthetic evaluations and purchase intentions (Hussain, 2025). Within this face-perception framework, the eye region plays a uniquely critical role. The mere presence of eyes—even in highly schematic form—is often sufficient to trigger face detection, face-like perception, and related attributions of agency or emotional expression (Perea-García et al., 2025). Indeed, research has shown that simply adding eyes to an otherwise inanimate object can induce perceptions of agency and emotional expression. Beyond mere presence, specific ocular features such as pupil size, iris brightness, and scleral visibility have been shown to influence perceived cuteness and approachability. Perea-García et al. (2025) demonstrated that pupil size and iris brightness interact to affect affective responses (Konno et al., 2023), while Konno et al. (2023) found that darker irises in domesticated dogs are perceived as more juvenile and friendly compared to the lighter irises of wolves (Brown, 2010). These findings underscore that eyes are not merely one among many facial features, but a uniquely potent channel for social and emotional signaling. In mascot design, the configuration of eyes may therefore interact holistically with infantile body proportions to shape attentional responses—a premise that motivates the configurational approach adopted in the present study. Brown (2010) similarly noted that adding eyes is one of the simplest ways to make objects appear face-like and support anthropomorphic interpretation (Williams and Castelhano, 2019). Their findings revealed that the attentional prominence of animal mascots is closely tied to their physical and psychological proximity to humans. Bipedal animals capable of upright walking emerged as the preferred choice for brand mascots.

Based on these theoretical considerations, the present study focused on three design variables: mascot type, head-to-body ratio, and eye size. These variables were selected because they correspond to different levels of mascot design: character category, body proportion, and facial-feature configuration. Mascot type was included because Human, Animal, and Robot mascots represent different degrees and forms of face-likeness, biological familiarity, and anthropomorphic interpretation. Human mascots are closest to real human faces and may therefore rely more strongly on canonical facial cues related to face perception (Tong et al., 2000; Windhager et al., 2008). Animal mascots combine non-human novelty with biologically familiar juvenile features, making them relevant to baby-schema responses beyond human faces (Borgi et al., 2014). Robot mascots represent artificial agents that may contain simplified face-like structures without biological identity, allowing the study to examine whether face-like and infantile cues operate similarly in technological or non-biological characters (Waytz et al., 2014; Miesler et al., 2011). Head-to-body ratio was selected as a body-proportion indicator related to baby-schema cues. Baby schema research emphasizes that relatively large heads, enlarged eyes, round faces, and compact infant-like proportions can influence cuteness perception, gaze allocation, and preference (Lorenz, 1943; Glocker et al., 2009; Borgi et al., 2014; Kanwisher et al., 1997). Therefore, in the present study, head-to-body ratio was used as a controllable design proxy for compact infant-like body proportion. Eye size was selected as an ocular cue relevant to both baby schema and face-like perception. The eye region is central to face perception and social interpretation, and eye-related features can influence perceived agency, emotional expression, cuteness, and approachability (Perea-García et al., 2025; Konno et al., 2023; Brown, 2010). Together, these three variables allowed the study to examine mascot design at the levels of type, body proportion, and eye-related facial cues.

Eye-tracking technology measures eye movements, determining an individual's attentional focus by identifying fixation points and recording the temporal and spatial aspects of visual processing. It provides valuable insights into scene perception and visual behavior (Graham et al., 2012). As a direct means of obtaining behavioral data, eye tracking captures individuals' fixation patterns in real time, making it particularly useful for studying consumer attention (Chaudhuri et al., 2021; Najemnik and Geisler, 2005). Eye movement data is both applicable and objective for visual quality assessment, effectively compensating for biases inherent in subjective perception (Liu et al., 2019). Key metrics recorded by eye-tracking devices include first fixation time, first fixation duration, total fixation duration, visit duration, fixation count, and visit count (Ilhan and Togay, 2023). Fixation, defined as the period of relative eye stillness during visual processing, is closely associated with cognitive processing (Goldberg et al., 2021). Fixation duration, in particular, serves as an established measure of attentional allocation toward a specific object or area (Holmqvist et al., 2011). Research has demonstrated that fixation duration is influenced by both stimulus properties and task demands, reflecting the degree of attentional engagement. Fixation duration and dwell time on an area of interest are widely used indicators of visual-attentional allocation in eye-tracking research. Under a defined viewing task, longer fixation duration on a stimulus generally indicates greater allocation of visual processing resources to that stimulus, although fixation measures should be interpreted in relation to task demands and stimulus context (Orquin and Mueller Loose, 2013; Kim, 2017).

ANOVA is useful for testing mean differences and interaction effects among experimentally manipulated factors (Olan et al., 2016). However, fsQCA addresses a different and complementary question: whether different combinations of conditions can form multiple sufficient pathways to the same outcome. This configurational approach is particularly useful for design research, where high visual attention may arise from different feature combinations rather than from a single universal optimum (Xi et al., 2016).

The objectives of this study were threefold. First, Experiments 1–3 examined how mascot type, head-to-body ratio, and eye size were associated with fixation-based visual attention. Second, a questionnaire-based preference rating study examined whether the attention patterns observed in Experiments 1–3 converged with self-reported liking. Third, Experiment 4 adopted a full-factorial stimulus structure, and fsQCA was used to identify configurational pathways associated with high total fixation duration.

## 2 Materials and methods

### 2.1 Overall research design and stimulus design

The study followed a three-stage design. First, Experiments 1–3 examined the effects of each design variable on fixation-based visual attention: Experiment 1 focused on mascot type, Experiment 2 focused on head-to-body ratio, and Experiment 3 focused on eye size. Second, a questionnaire-based preference rating study examined whether the attention patterns observed in Experiments 1–3 converged with self-reported liking. Third, Experiment 4 adopted a full-factorial stimulus structure and provided the eye-tracking data for fsQCA, which was used to identify combinations of mascot type, head-to-body ratio, and eye size associated with high total fixation duration.

Across Experiments 1–3, the stimulus sets were designed to control the target variable as far as possible within each experimental context. In Experiment 1, mascot type was the primary manipulated variable, while eye size and head-to-body ratio were kept as consistent as possible, and all stimuli were presented in grayscale. In Experiment 2, head-to-body ratio was systematically varied across the four ratio conditions within each mascot type, while eye size was kept at a medium level and all stimuli were presented in grayscale. In Experiment 3, eye size was varied across large, medium, and small conditions within each mascot type, while head-to-body ratio was kept at 1:2 and all stimuli were presented in grayscale.

#### 2.1.1 Mascot type

Experiment 1 examined the influence of mascot type on fixation-based visual attention. The experimental design included six images, each representing one of three mascot types: Human, Animal, and Robot, as illustrated in Table 1. Each type was represented by two images. The stimuli were presented in grayscale, and eye size and head-to-body ratio were kept as consistent as possible across mascot types. For gaze-data extraction, fixation metrics were calculated separately for the screen region occupied by each stimulus image in the displayed set. These regions corresponded to individual stimulus images and were used only for extracting fixation metrics, not as experimental variables. Participant gender was recorded and included only as an exploratory between-subjects demographic variable rather than as a manipulated stimulus variable.

**Table 1:**
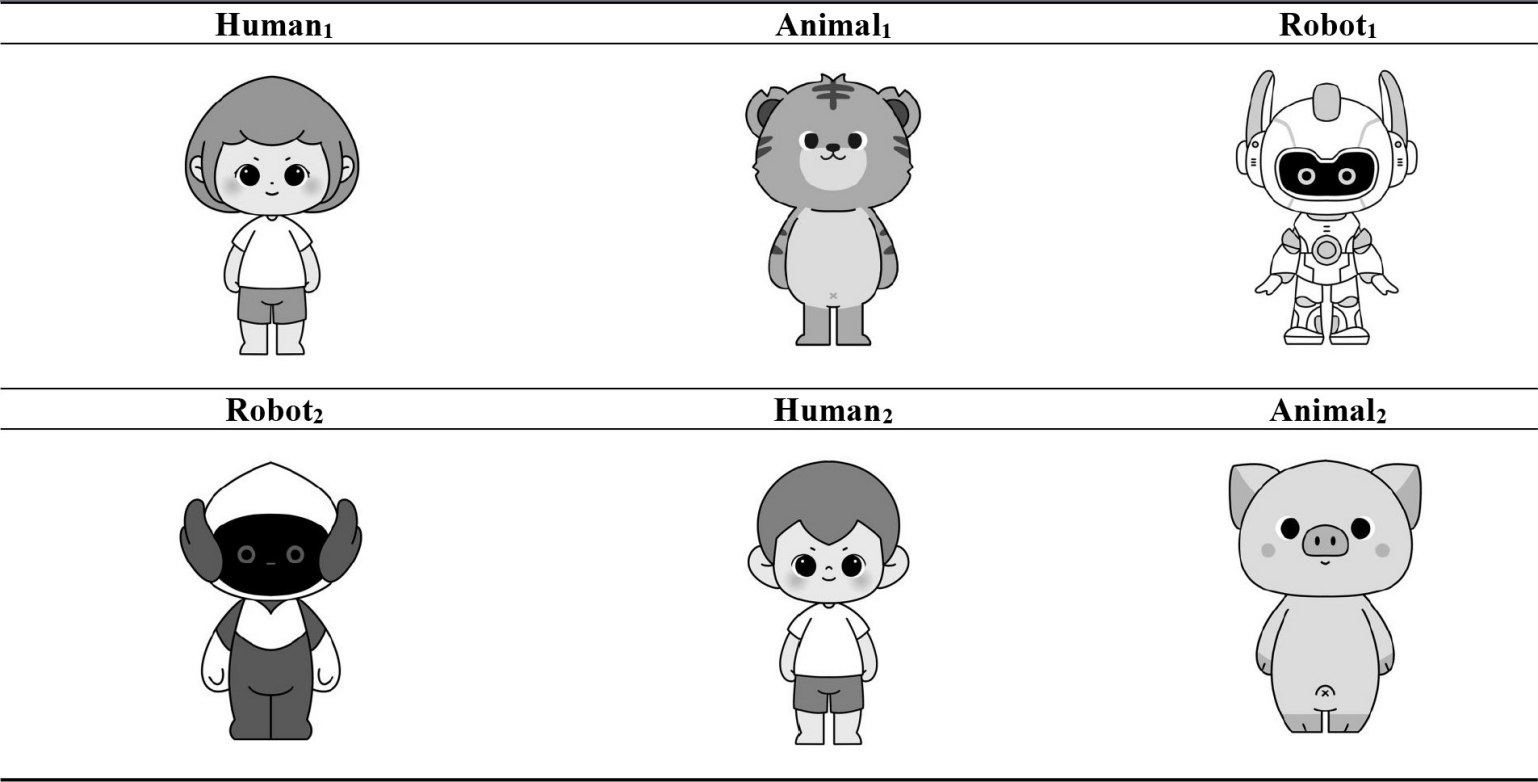
Stimuli used in Experiment 1 by mascot type.

#### 2.1.2 Head-to-body ratio

**Figure 1:**
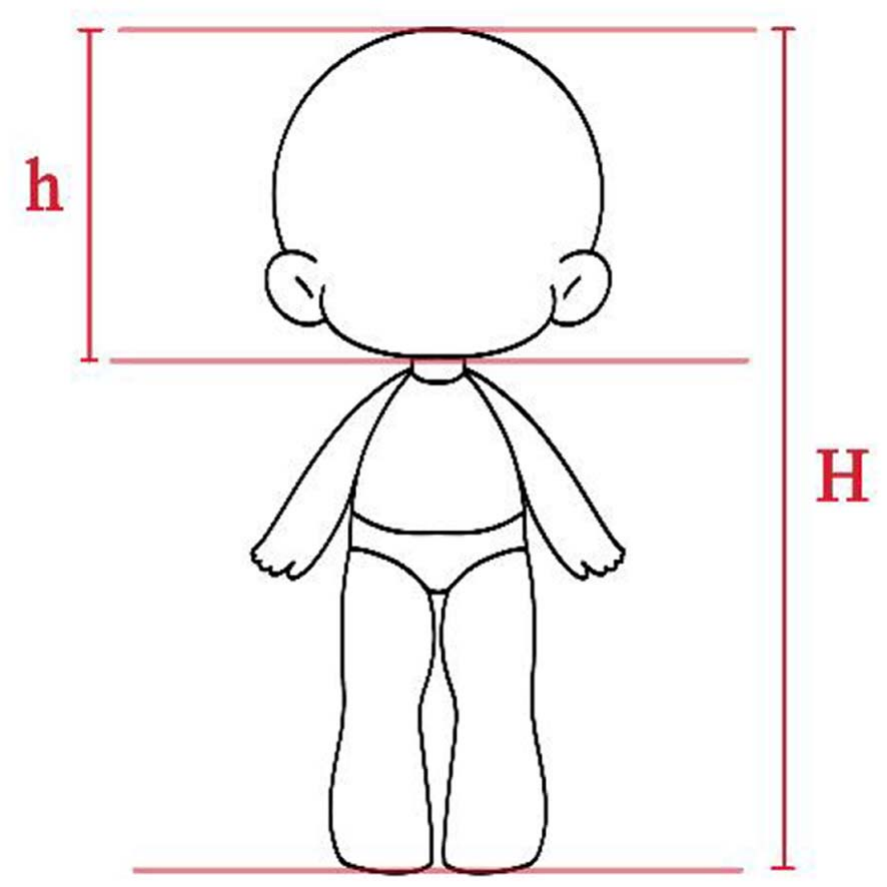
Definition of head-to-body ratio. In this study, the ratio was expressed as head height (h) to total body height (H), with head height set as 1. Thus, 1:1.5 indicates a larger relative head size and a more compact body proportion, whereas 1:3 indicates a smaller relative head size and a more elongated body proportion.

Experiment 2 examined the influence of head-to-body ratio on fixation-based visual attention within each mascot type. In this study, head-to-body ratio was expressed as the ratio of head height(h) to total body height(H) (Barbosa et al., 2021), with head height set as 1 (Figure 1). The experimental design followed a 3 mascot type × 4 head-to-body ratio structure, including Human, Animal, and Robot mascots at four ratio levels: 1:1.5, 1:2, 1:2.5, and 1:3, as illustrated in Table 2. These four levels were selected during stimulus development to represent a graded range from compact, large-head proportions to more elongated body proportions. In this notation, 1:1.5 indicates the largest relative head size and the most compact body proportion, whereas 1:3 indicates the smallest relative head size and the most elongated body proportion. Eye size was kept at a medium level as far as possible, and all stimuli were presented in grayscale. For gaze-data extraction, fixation metrics were calculated separately for the screen region occupied by each stimulus image in the displayed set. These regions corresponded to individual stimulus images and were used only for extracting fixation metrics, not as experimental variables.

**Table 2:**
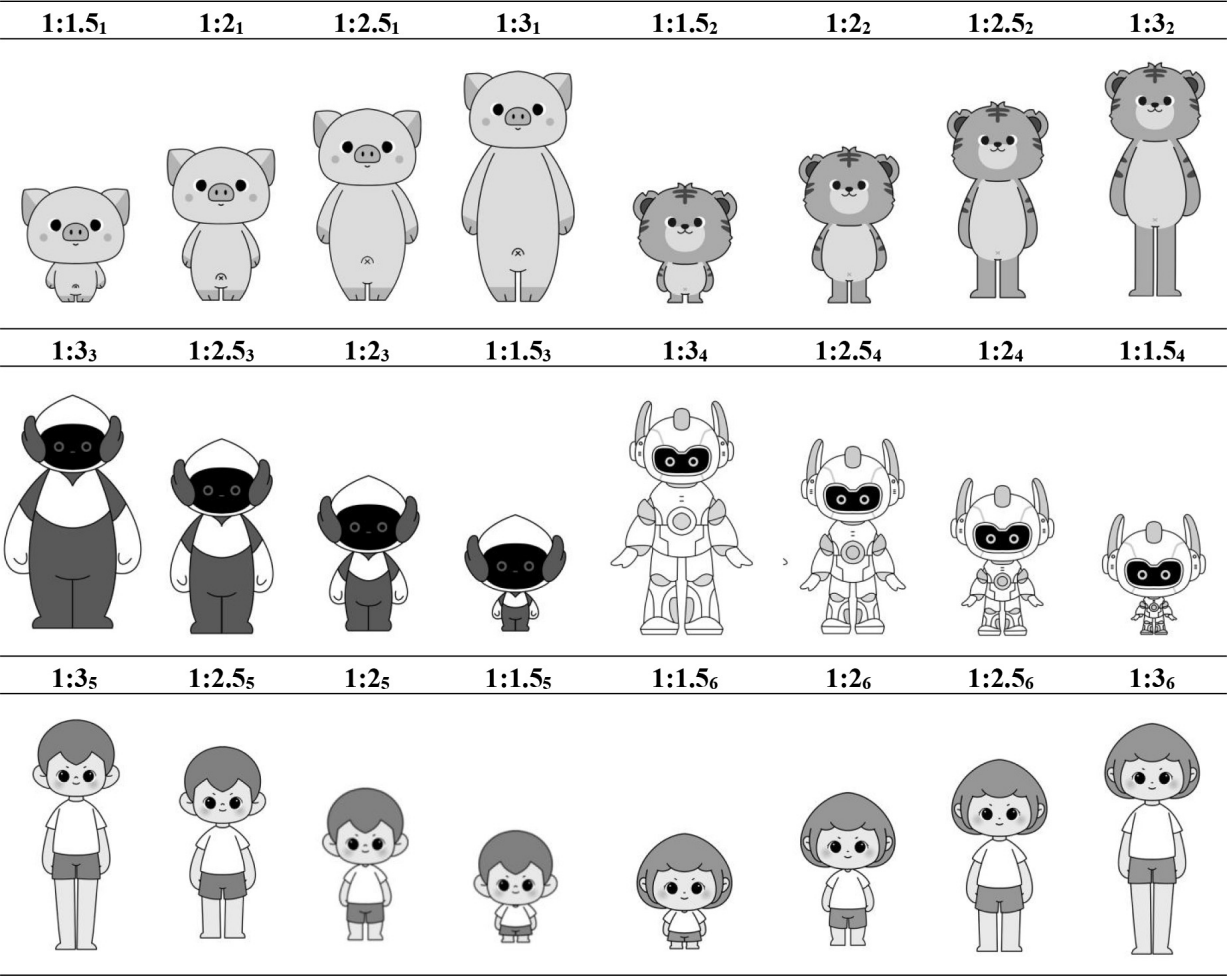
Stimuli used in Experiment 2 by head-to-body ratio.

#### 2.1.3 Eye size

Experiment 3 examined the influence of eye size on fixation-based visual attention within each mascot type. The experimental design followed a 3 mascot type × 3 eye size structure, including Human, Animal, and Robot mascots with large, medium, and small eyes, as illustrated in Table 3. Head-to-body ratio was kept at 1:2 as far as possible, and all stimuli were presented in grayscale. Because changes in eye size may also alter perceived eye contour, contrast, and expressiveness, the eye-size manipulation is interpreted as an ocular-feature manipulation rather than as a purely geometric size manipulation. For gaze-data extraction, fixation metrics were calculated separately for the screen region occupied by each stimulus image in the displayed set. These regions corresponded to individual stimulus images and were used only for extracting fixation metrics, not as experimental variables.

**Table 3:**
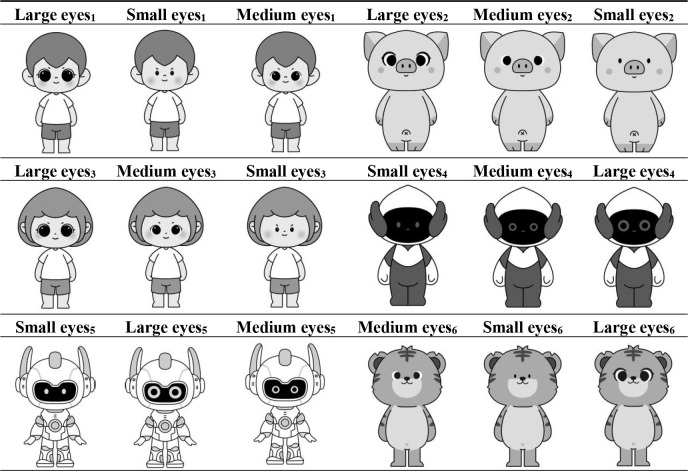
Stimuli used in Experiment 3 by eye-size condition.

### 2.2 Participants

A total of 290 young Chinese adults were recruited from a university context to participate in the study. The sample included 145 males and 145 females, with an overall mean age of 22.88 years (SD = 4.45, range = 18–35 years). Young adults were selected because they are a relevant audience for contemporary brand mascot communication, particularly in digital media, e-commerce, and social media contexts where mascots are frequently used to attract attention and support brand engagement. Independent participant samples were used across the study components to avoid habituation and repeated-exposure effects. Experiments 1–3 each included 30 participants, the questionnaire-based preference rating study included 100 participants, and Experiment 4 included another independent sample of 100 participants. No participant took part in more than one study component. The demographic information for each study component is summarized in Table 4. All participants met the following inclusion criteria: corrected or uncorrected visual acuity of at least 1.0 in both eyes, no astigmatism, and no other self-reported vision impairments. Before participation, all participants provided informed consent.

**Table 4:** Demographic information of participants by study component.

Study component	*N*	Male	Female	Age M	Age SD	Age range
Experiment 1	30	15	15	21.67	4.35	18–27
Experiment 2	30	15	15	21.43	3.98	18–26
Experiment 3	30	15	15	23.56	4.55	18–28
Preference rating study	100	50	50	23.33	3.87	18–35
Experiment 4 / fsQCA	100	50	50	23.02	5.01	18–35
Total	290	145	145	22.88	4.45	18–35

Independent participant samples were used across all study components. Experiment 4 provided the eye-tracking data used for fsQCA.

### 2.3 Experimental equipment and environment

**Figure 2:**
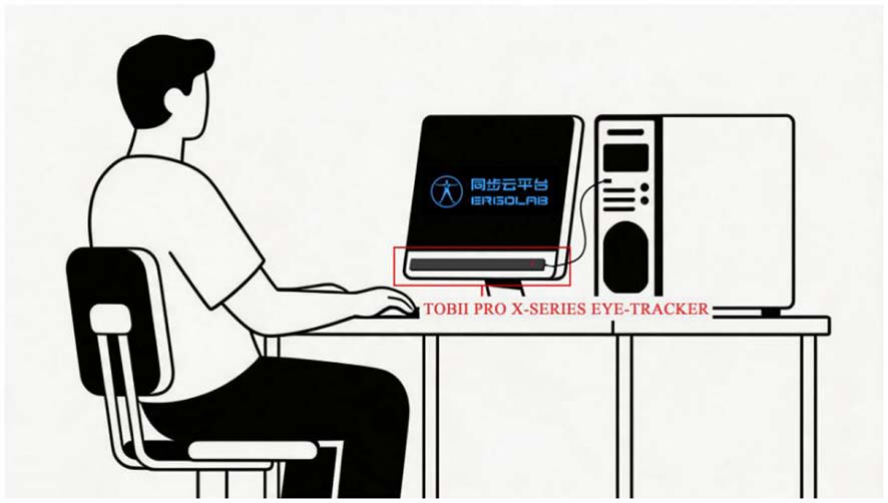
The eye tracker system for the experiment.

The experiment was conducted in the Human Factors and Ergonomics Laboratory at Nanjing Forestry University. The experimental setup, as shown in Figure 2, consisted of a DELL OptiPlex 7000 host computer running ErgoLAB 3.0 software for stimulus presentation, experimental control, and eye-tracking data collection. Stimuli were displayed on a DELL E2423H monitor (53.15 cm × 29.90 cm; resolution 1,920 × 1,080 pixels). Eye movements were recorded using a Tobii Pro X-Series eye-tracker positioned at the base of the monitor. Participants were seated approximately 70 cm from the screen. The laboratory environment was maintained at a stable temperature and humidity level (25 °C, 50%) to ensure participant comfort and minimize potential environmental influences on the results. A schematic diagram of the experimental setup is provided in Figure 2.

### 2.4 Experimental process

A pilot study was conducted with three participants (separate from the main sample) to determine the appropriate viewing duration for each stimulus and to verify the clarity of the instructional text. Participants confirmed that approximately 30 s per stimulus was sufficient for thorough observation, and that the instructional text effectively guided their attention to the designated design features. During the pilot study, unstructured free viewing led participants to attend to different stimulus attributes according to their individual interests, which made it difficult to compare attention to the intended design variable. Therefore, feature-specific instructions were used to standardize the evaluation context across participants. Prior to each experiment, participants completed a standard eye-tracker calibration procedure while seated approximately 70 cm from the monitor.

During the experiment, instructional text was displayed on screen for each phase, guiding participants to focus on the specific design feature under investigation:

“Please view the type of mascot you find most interesting.”

“Please view the head-to-body ratio of the mascot you find most interesting.”

“Please view the eye size of the mascot you find most interesting.”

At the beginning of each experimental phase, an instructional prompt corresponding to the target design variable was displayed. Specifically, participants were instructed to focus on mascot type, head-to-body ratio, or eye size depending on the experiment. The instruction did not vary across individual stimulus images within the same phase. After the instruction, the full stimulus set for that phase was presented simultaneously on the screen for 30 s. This duration was determined by the pilot study as sufficient for participants to inspect all stimulus images within the set. This goal-directed viewing paradigm was adopted to examine how design features guide visual attention when viewers consciously evaluate mascots. In Experiment 1, six stimulus images were arranged in a 3 × 2 grid layout. In Experiment 2, 24 stimulus images were arranged in a 8 × 3 grid layout. In Experiment 3, 18 stimulus images were arranged in a 6 × 3 grid layout. Stimulus positions were fixed across participants to maintain a consistent display structure. For gaze-data extraction, areas of interest (AOIs) were defined as the rectangular screen regions occupied by each stimulus image within the displayed set. Fixation metrics were calculated separately for each AOI. These AOIs corresponded to individual stimulus images and were used only to extract fixation metrics, not as experimental variables or labels.

Eye-tracking data were exported from ErgoLAB 3.0 and organized in Microsoft Excel. For each stimulus image, the following metrics were compiled: fixation count (FC), fixation duration (FD), total fixation count (TFC), and total fixation duration (TFD). Gender was also recorded for between-subjects analyses. Data were analyzed using SPSS 27.0 and visualized using Origin 2022. Tables 5 and 6 summarize the independent variables and eye-tracking measures used in the study.

**Table 5:** Independent variables and their levels.

Category	Variable	Levels	Role in analysis
Manipulated design variable	Mascot type	Human, Animal, Robot	Within-subjects factor
Manipulated design variable	Head-to-body ratio	1:1.5, 1:2, 1:2.5, 1:3	Within-subjects factor
Manipulated design variable	Eye size	Large, Medium, Small	Within-subjects factor
Participant-level variable	Gender	Male, Female	Exploratory between-subjects demographic variable

Participant gender was not a manipulated stimulus variable and was included only for exploratory demographic comparisons.

**Table 6:** Eye-tracking data and measurement indicators.

Eye tracker data	Fixation count	Fixation duration	Total fixation count	Total fixation duration
Abbreviation	FC	FD	TFC	TFD
Implication	Number of fixations recorded for a stimulus image.	Duration of fixations allocated to a stimulus image; used as the main dependent variable in Experiments 1–3.	Sum of fixation counts across participants or stimuli, used descriptively.	Sum of fixation duration; used as the outcome condition in the fsQCA analysis.

For Experiments 1–3, fixation duration (FD) was used as the primary dependent variable for inferential analysis. In Experiment 1, FD was averaged within each mascot type for each participant and analyzed using a one-way repeated-measures ANOVA with mascot type as the within-subjects factor. In Experiment 2, FD was analyzed using a 3 mascot type × 4 head-to-body ratio repeated-measures ANOVA. In Experiment 3, FD was analyzed using a 3 mascot type × 3 eye size repeated-measures ANOVA. Participant gender was included only as an exploratory between-subjects demographic variable, rather than as a manipulated stimulus variable, and gender-related results were interpreted cautiously.

For the repeated-measures ANOVAs, the assumption of sphericity was tested using Mauchly's test. When the assumption of sphericity was met, uncorrected degrees of freedom were reported. When sphericity was violated, Greenhouse–Geisser corrected degrees of freedom and *p-values* were reported. Bonferroni-corrected pairwise comparisons were conducted following significant main effects, and simple-effect analyses were conducted when interactions were significant. Effect sizes were reported as partial eta squared (ηp^2^), and the significance level was set at α = 0.05.

Because Experiments 1–3 used separate participant samples and different stimulus sets, effect sizes from these models were not used to establish a direct hierarchy of influence among the three design factors. ANOVA was used to test mean differences and interaction effects, whereas fsQCA addressed a complementary configurational question: whether different combinations of mascot type, head-to-body ratio, and eye size formed sufficient pathways associated with high total fixation duration. Therefore, in Experiment 4, total fixation duration (TFD) was used as the fsQCA outcome to examine configurational patterns of visual attention.

### 2.5 Subjective preference rating study

To complement the eye-tracking results and to distinguish fixation-based visual attention from subjective preference, an additional questionnaire-based preference rating study was conducted. This study aimed to examine whether the visual-attention patterns observed in Experiments 1–3 were convergent with young adults' self-reported preferences for the corresponding mascot design features.

A total of 100 young adults were recruited for the questionnaire study. This sample was independent from the eye-tracking samples used in Experiments 1–3 and Experiment 4. All participants reported normal or corrected-to-normal vision and provided informed consent before participation. The questionnaire used the same mascot stimuli as Experiments 1–3, including the mascot type stimuli, head-to-body ratio stimuli, and eye-size stimuli. The stimuli were presented in three sections corresponding to the three design variables: mascot type, head-to-body ratio, and eye size. Within each section, the order of stimulus presentation was randomized to reduce order effects.

Participants rated each stimulus using a 7-point Likert scale. The rating item was: “How much do you like this mascot design?” The response options ranged from 1 = strongly dislike to 7 = strongly like. For each participant, preference ratings were averaged within each condition. Specifically, ratings were averaged across Human, Animal, and Robot mascots for mascot type; across the four head-to-body ratio conditions of 1:1.5, 1:2, 1:2.5, and 1:3; and across the three eye-size conditions of large eyes, medium eyes, and small eyes.

The preference rating study was designed to validate the single-variable patterns observed in Experiments 1–3 rather than to test full-factorial preference interactions. Therefore, three repeated-measures ANOVAs were conducted separately for mascot type, head-to-body ratio, and eye size. To reduce the risk of familywise Type I error across the three omnibus tests, Holm–Bonferroni correction was applied. Bonferroni-corrected pairwise comparisons were used for *post hoc* tests where appropriate. The questionnaire results were interpreted as subjective preference ratings, whereas the eye-tracking results were interpreted as fixation-based visual attention.

### 2.6 Combinatorial experiment and fsQCA analysis

#### 2.6.1 Combinatorial experiment design

To examine how mascot design features operate in combination, a fourth eye-tracking experiment was conducted using an independent participant sample. This experiment focused on three design variables: mascot type, head-to-body ratio, and eye size. Mascot type included three levels: Human-like mascot, Animal mascot, and Robot mascot; head-to-body ratio included four levels: 1:1.5, 1:2, 1:2.5, and 1:3; and eye size included three levels: large, medium, and small. A full-factorial design was adopted, resulting in 36 stimulus combinations.

A total of 100 young adults participated in this experiment. This sample was independent from Experiments 1–3 and from the questionnaire-based preference rating study. To reduce fatigue, the 36 stimuli were divided into three blocks, with 12 stimuli in each block. Within each block, the 12 stimuli were arranged in a 4 × 3 grid layout. The order of the three blocks was fixed across participants. Participants completed the three blocks sequentially, with short rest intervals between blocks. At the beginning of each block, participants were presented with the instruction: “Please freely view the mascot you find most visually interesting.” The dependent variable in the fsQCA analysis was total fixation duration; therefore, the configurational results are interpreted as patterns of fixation-based visual attention rather than as direct measures of subjective appeal.

#### 2.6.2 fsQCA method

Data analysis was conducted using fsQCA 3.0. The fsQCA analysis was used to examine whether different combinations of mascot type, head-to-body ratio, and eye size were associated with high total fixation duration (TFD). In this study, TFD was selected as the outcome condition because it reflects the overall level of fixation-based visual attention allocated to each stimulus configuration.

Before conducting the fsQCA analysis, the raw data were calibrated into fuzzy-set membership scores. For the outcome condition, the 75th, 50th, and 25th percentiles of TFD were used as the three calibration anchors, corresponding to full membership, the crossover point, and full non-membership, respectively. After calibration, higher membership scores indicated a stronger degree of membership in the set of high-TFD configurations (Kraus et al., 2018).

The antecedent conditions consisted of three mascot design variables: mascot type, head-to-body ratio, and eye size. Mascot type included Human, Animal, and Robot mascots; head-to-body ratio was calibrated as a compact-ratio condition; and eye size included large, medium, and small eye conditions. For the fsQCA analysis, ratios of 1:1.5 and 1:2 were coded as membership in the compact-ratio set because they represented relatively large-head, compact body proportions, whereas ratios of 1:2.5 and 1:3 were coded as non-membership because they represented more elongated body proportions. This coding was used to align the fsQCA condition with the theoretical distinction between stronger and weaker baby-schema-like body proportions. These variables were coded according to the experimental design and then entered into the fsQCA procedure.

The fsQCA analysis included necessity analysis and sufficiency analysis. Necessity analysis was first conducted to determine whether any single condition was consistently present in cases with high TFD. Following conventional fsQCA criteria, the consistency threshold for necessary conditions was set at 0.90 (Beynon et al., 2016). Sufficiency analysis was then conducted using the truth-table algorithm to identify configurations of design conditions associated with high TFD. The consistency threshold for sufficiency analysis was set at 0.80, and the frequency threshold was set at 1 (Epley et al., 2007).

In the interpretation of the results, conditions appearing in the identified configurations were described as core or peripheral conditions according to the fsQCA output. A core condition was understood as a central element within a sufficient configuration, rather than as a necessary condition unless it also met the necessity threshold.

## 3 Results

### 3.1 Attention to mascot type

**Figure 3:**
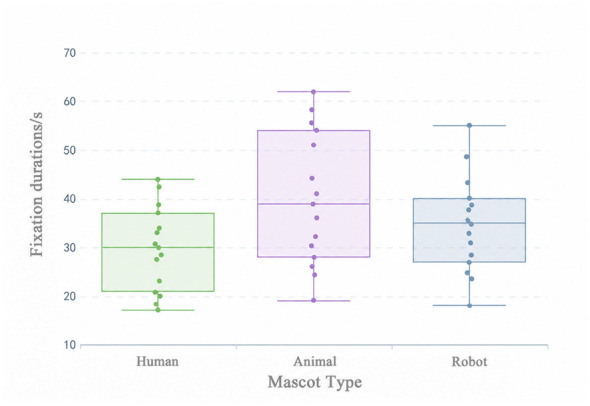
Box plot of fixation duration by mascot type.

**Figure 4:**
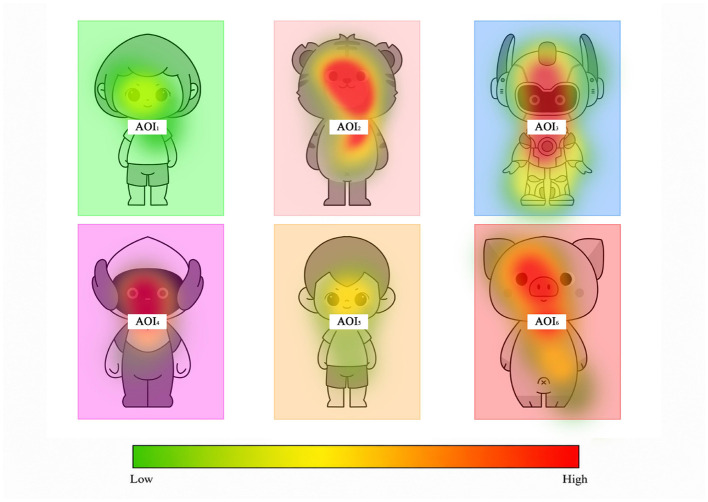
Heat map showing the spatial distribution of fixation allocation across mascot-type stimuli.

Experiment 1 examined whether fixation-based visual attention differed across three mascot types: Human, Animal, and Robot. Thirty young adults participated in this experiment. For each participant, fixation duration was averaged across stimuli within each mascot type and then entered into the repeated-measures ANOVA. Mauchly's test indicated that the assumption of sphericity was met, W = 0.92, χ^2^(2) = 2.26, *p* = 0.32; therefore, uncorrected degrees of freedom are reported. The repeated-measures ANOVA results showed a significant effect of mascot type on fixation duration, *F*_(2,58)_ = 3.53, *p* = 0.04, η_p^2^ = 0.11, as shown in Table 7 and Figures 3, 4.

**Table 7:** Eye-tracking results for mascot type.

Analysis section	Effect / condition	*F*	*p*	ηp^2^	TFC	TFD(s)	Mean FD (s)	SD of FD
ANOVA	Mascot type	3.53	0.04	0.11				
Descriptive	Human				350	145.70	12.14	4.45
Descriptive	Animal				502	280.33	23.36	16.98
Descriptive	Robot				402	172.95	14.41	7.09

Bonferroni-corrected pairwise comparisons were conducted to further examine differences among the three mascot types. The results showed that Animal mascots received significantly longer fixation durations than Human mascots, *p* = 0.038. However, the differences between Animal and Robot mascots, *p* = 0.112, and between Robot and Human mascots, *p* = 0.741, were not statistically significant.

The fixation duration differed across the three mascot types. Animal mascots received the longest fixation duration (M = 23.36 s, SD = 16.98), followed by Robot mascots (M = 14.41 s, SD = 7.09) and Human mascots (M = 12.14 s, SD = 4.45). The same pattern was observed for total fixation duration, with Animal mascots showing the highest TFD value (280.33 s), followed by Robot mascots (172.95 s) and Human mascots (145.70 s). These results indicate that, under the present viewing task, Animal mascots were associated with the highest level of fixation-based visual attention among the three mascot types.

Exploratory participant-gender comparisons showed no significant differences in fixation duration across mascot types, all *ps* > 0.05. Because participant gender was not a manipulated stimulus variable, these results were interpreted only as demographic comparisons.

Overall, the fixation-based attention pattern for mascot type followed the order Animal > Robot > Human. This result suggests that mascot type influenced visual-attentional allocation, with Animal mascots receiving the strongest fixation-based attention in this experiment.

### 3.2 Attention to mascot head-to-body ratio

Experiment 2 examined whether fixation-based visual attention differed across four head-to-body ratios within each mascot type. The analysis used a 3 mascot type × 4 head-to-body ratio repeated-measures ANOVA on fixation duration. Thirty young adults participated in this experiment. For each participant, fixation duration was averaged within each mascot type × head-to-body ratio condition and then entered into the repeated-measures ANOVA.

**Figure 5:**
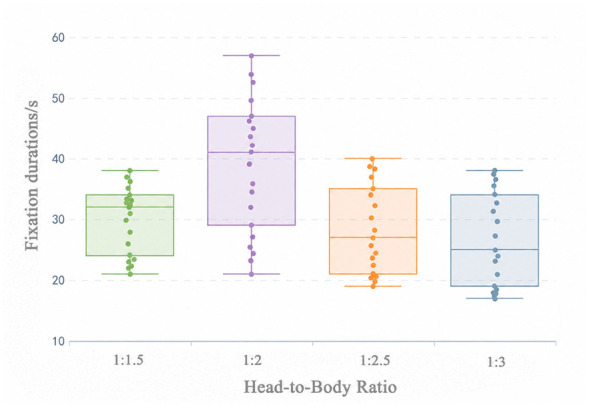
Box plot of fixation duration by head-to-body ratio.

**Figure 6:**
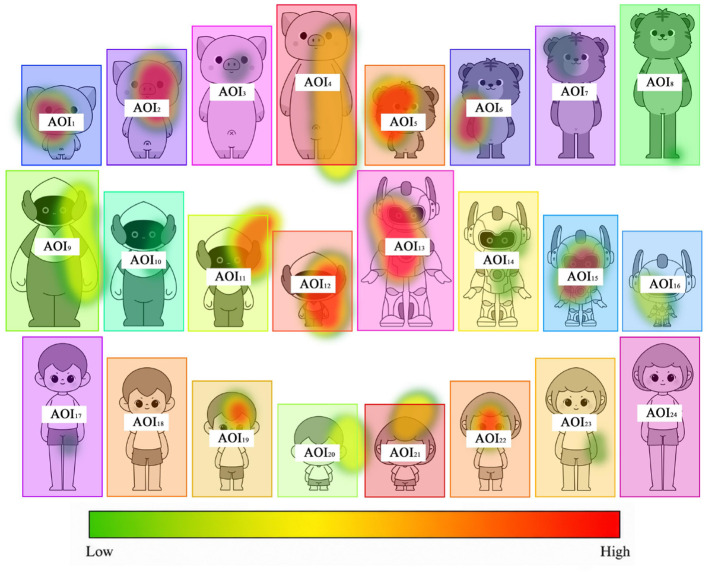
Heat map showing the spatial distribution of fixation allocation across head-to-body-ratio stimuli.

The repeated-measures ANOVA showed a significant main effect of mascot type, *F*_(2,58)_ = 4.62, *p* = 0.014, ηp^2^ = 0.14. The main effect of head-to-body ratio was also significant, *F*_(3,87)_ = 4.05, *p* = 0.010, ηp^2^ = 0.12. The mascot type × head-to-body ratio interaction was not significant, *F*_(6,174)_ = 1.21, *p* = 0.304, ηp^2^ = 0.04, as shown in Table 8 and Figures 5, 6. Because the interaction was not significant, the main effect of head-to-body ratio was interpreted across mascot types. Bonferroni-corrected pairwise comparisons showed that the 1:2 ratio received significantly longer fixation durations than 1:1.5, 1:2.5, and 1:3.

**Table 8:** Eye-tracking results for head-to-body ratio.

Analysis section	Effect / condition	*F*	*p*	ηp^2^	TFC	TFD(s)	Mean FD (s)	SD of FD
ANOVA	Mascot type	4.62	0.014	0.14				
ANOVA	Head-to-body ratio	4.05	0.010	0.12				
ANOVA	Mascot type × head-to-body ratio	1.21	0.304	0.04				
Descriptive	1:1.5				184	167.65	16.77	9.73
Descriptive	1:2				388	297.41	29.74	17.73
Descriptive	1:2.5				300	151.30	15.13	8.24
Descriptive	1:3				300	148.32	14.83	7.70

Descriptively, fixation duration varied across the four head-to-body ratios. Mascots with a 1:2 head-to-body ratio received the longest fixation duration (M = 29.74 s, SD = 17.73), followed by mascots with ratios of 1:1.5 (M = 16.77 s, SD = 9.73), 1:2.5 (M = 15.13 s, SD = 8.24), and 1:3 (M = 14.83 s, SD = 7.70). Descriptively, the same pattern was observed for total fixation duration, with the 1:2 ratio showing the highest TFD value (297.41 s), followed by 1:1.5 (167.65 s), 1:2.5 (151.30 s), and 1:3 (148.32 s). Because the mascot type × head-to-body ratio interaction was not significant, this pattern suggests that the attentional advantage of the 1:2 ratio was relatively consistent across mascot types.

Exploratory participant-gender comparisons showed no significant differences in fixation duration across the head-to-body ratio conditions, all ps > 0.05. These results were interpreted only as demographic comparisons because participant gender was not a manipulated stimulus variable.

Overall, Experiment 2 showed that head-to-body ratio was associated with fixation-based visual attention. The 1:2 ratio showed the strongest descriptive attention pattern, while the non-significant mascot type × head-to-body ratio interaction indicated that the effect of head-to-body ratio did not differ significantly across mascot types.

### 3.3 Attention to mascot eye size

Experiment 3 examined whether fixation-based visual attention differed across three eye-size conditions within each mascot type. The analysis used a 3 mascot type × 3 eye size repeated-measures ANOVA on fixation duration. Thirty young adults participated in this experiment. For each participant, fixation duration was averaged within each mascot type × eye size condition and then entered into the repeated-measures ANOVA.

**Figure 7:**
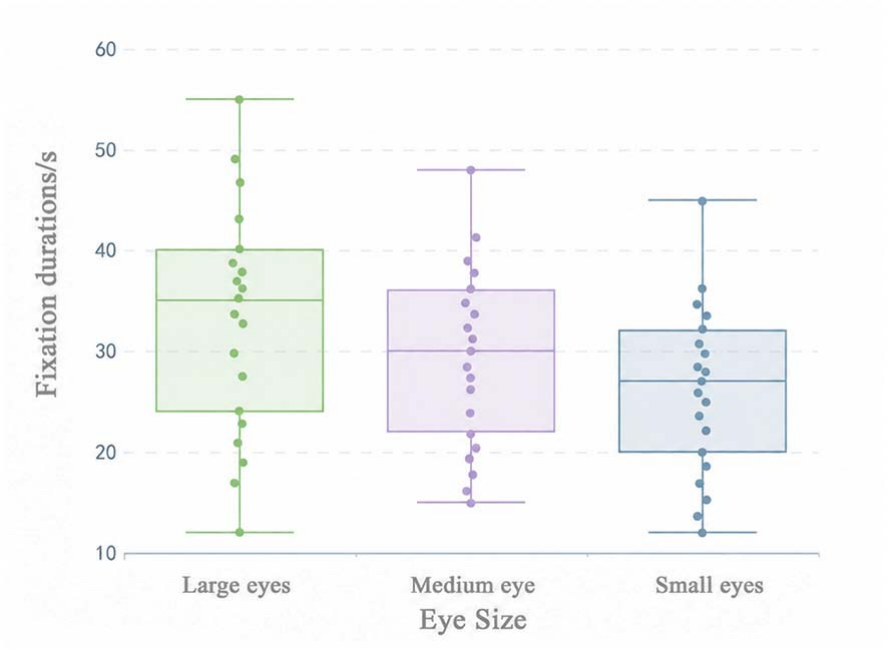
Box plot of fixation duration by eye size.

**Figure 8:**
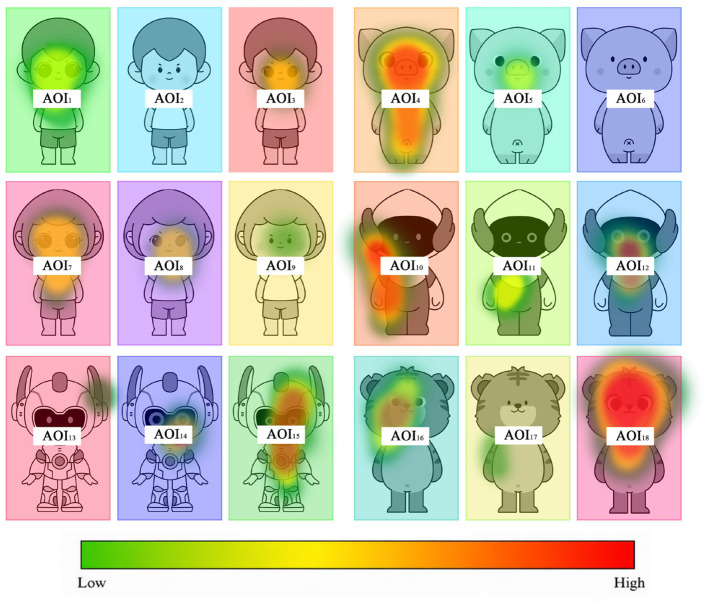
Heat map showing the spatial distribution of fixation allocation across eye-size stimuli.

The repeated-measures ANOVA showed a significant main effect of mascot type, *F*_(2,58)_ = 4.38, *p* = 0.017, ηp^2^ = 0.13. The main effect of eye size was significant, *F*_(2,58)_ = 3.72, *p* = 0.030, ηp^2^ = 0.11. The mascot type × eye size interaction was significant, *F*_(4,116)_ = 2.81, *p* = 0.029, ηp^2^ = 0.09, as shown in Table 9 and Figures 7, 8. Because the mascot type × eye size interaction was significant, simple-effect analyses were conducted to examine the effect of eye size separately within each mascot type. The simple effect of eye size was significant for Human mascots, *F*_(2,58)_ = 5.26, *p* = 0.008, ηp^2^ = 0.15, but not significant for Animal mascots, *F*_(2,58)_ = 1.21, *p* = 0.305, ηp^2^ = 0.04, or Robot mascots, *F*_(2,58)_ = 0.94, *p* = 0.396, ηp^2^ = 0.03. This indicates that the effect of eye size was most pronounced for Human mascots, whereas differences among eye-size conditions were weaker for Animal and Robot mascots.

**Table 9:** Eye-tracking results for eye size.

Analysis section	Effect / condition	*F*	*p*	ηp^2^	TFC	TFD(s)	Mean FD (s)	SD of FD
ANOVA	Mascot type	4.38	0.017	0.13				
ANOVA	Eye size	3.72	0.030	0.11				
ANOVA	Mascot type × Eye size	2.81	0.029	0.09				
Descriptive	Large eyes				363	342.05	25.66	15.14
Descriptive	Medium eyes				300	268.05	20.11	8.33
Descriptive	Small eyes				184	222.13	16.66	6.97

Descriptively, fixation duration varied across the three eye-size conditions. Mascots with large eyes received the longest fixation duration (M = 25.66 s, SD = 15.14), followed by mascots with medium eyes (M = 20.11 s, SD = 8.33) and small eyes (M = 16.66 s, SD = 6.97). Descriptively, the same pattern was observed for total fixation duration, with large-eye mascots showing the highest TFD value (342.05 s), followed by medium-eye mascots (268.05 s) and small-eye mascots (222.13 s).

Exploratory participant-gender comparisons showed no significant differences in fixation duration across eye-size conditions, all ps > 0.05.

Overall, Experiment 3 showed that eye size was associated with fixation-based visual attention, but the significant mascot type × eye size interaction indicated that the effect of eye size differed across mascot types. Therefore, the attentional role of eye size should be interpreted as type-contingent rather than uniform across all mascot categories.

### 3.4 Subjective preference rating validation

To further examine whether the fixation-based attention patterns were convergent with subjective preference, a questionnaire-based preference rating study was conducted with 100 young adults. Participants rated the mascot stimuli used in Experiments 1–3 on a 7-point Likert scale, with higher scores indicating stronger subjective preference. Because the questionnaire was organized into three sections corresponding to Experiments 1–3, and because its purpose was to validate single-variable preference patterns rather than configurational effects, three repeated-measures ANOVAs were conducted separately for mascot type, head-to-body ratio, and eye size. Holm–Bonferroni correction was applied across the three omnibus tests to control familywise Type I error. All three omnibus effects remained significant after correction. The subjective preference rating results are summarized in Table 10.

**Table 10:** Subjective preference ratings for mascot design features.

Design variable	Condition	Mean preference rating	SD	*F*	*p*	η_p^2^
Mascot type	Human	4.03	0.95	187.52	<0.001	0.65
	Animal	6.02	0.86			
	Robot	5.01	0.88			
Head-to-body ratio	1:1.5	5.51	0.90	452.89	<0.001	0.82
	1:2	6.49	0.80			
	1:2.5	4.00	0.92			
	1:3	2.49	0.84			
Eye size	Large eyes	6.00	0.87	406.33	<0.001	0.80
	Medium eyes	3.98	0.85			
	Small eyes	2.01	0.82			

For mascot type, Mauchly's test indicated that the assumption of sphericity was met, W = 0.97, χ^2^(2) = 2.98, *p* = 0.225; therefore, uncorrected degrees of freedom are reported. The repeated-measures ANOVA showed a significant effect on subjective preference ratings, *F*_(2,198)_ = 187.52, *p* < 0.001, η_p^2^ = 0.65. Bonferroni-corrected pairwise comparisons showed that Animal mascots received significantly higher preference ratings than Robot and Human mascots, and Robot mascots received significantly higher ratings than Human mascots, all *p* < 0.001.

For head-to-body ratio, Mauchly's test indicated that the assumption of sphericity was met, W = 0.92, χ^2^(5) = 8.02, *p* = 0.155; therefore, uncorrected degrees of freedom are reported. The repeated-measures ANOVA showed a significant effect on subjective preference ratings, *F*_(3,297)_ = 452.89, *p* < 0.001, η_p^2^ = 0.82. Bonferroni-corrected pairwise comparisons showed that the 1:2 ratio received significantly higher preference ratings than 1:1.5, 1:2.5, and 1:3, and all remaining pairwise differences were also significant, all *p* < 0.001.

For eye size, Mauchly's test indicated that the assumption of sphericity was met, W = 0.96, χ^2^(2) = 4.02, *p* = 0.134; therefore, uncorrected degrees of freedom are reported. The repeated-measures ANOVA showed a significant effect on subjective preference ratings, *F*_(2,198)_ = 406.33, *p* < 0.001, η_p^2^ = 0.80. Bonferroni-corrected pairwise comparisons showed that large eyes received significantly higher preference ratings than medium and small eyes, and medium eyes received significantly higher ratings than small eyes, all *p* < 0.001.

Overall, the subjective preference ratings showed a broadly convergent directional pattern with the eye-tracking results. Animal mascots, a 1:2 head-to-body ratio, and large eyes received both stronger fixation-based visual attention and higher subjective preference ratings. These findings suggest that the design features associated with longer fixation duration were also evaluated more favorably in the subjective preference ratings, while fixation duration and self-reported preference remain distinct measures. Thus, the questionnaire results complement the eye-tracking findings by showing a consistent pattern across attention-based and self-report measures.

### 3.5 fsQCA results: configurations associated with high total fixation duration

To examine the configurational effects among mascot design features, fuzzy-set Qualitative Comparative Analysis (fsQCA) was employed to identify configurations of antecedent conditions sufficient for high visual attention, measured by high total fixation duration (TFD). The three antecedent conditions were mascot type (human, animal, robot), head-to-body ratio (1:1.5, 1:2, 1:2.5, 1:3), and eye size (large, medium, small). Table 11 presents the raw TFD values and their calibrated fuzzy-set membership scores for all 36 design configurations.

**Table 11:** From raw TFD to calibrated scores for all design configurations.

Configuration No.	Mascot type	Head-to-body ratio	Eye size	TFD(s)	Calibrated TFD
1	Human	1:1.5	Large eyes	287.47	0.92
2	Human	1:1.5	Medium eyes	343.07	0.98
3	Human	1:1.5	Small eyes	215.52	0.59
4	Human	1:2	Large eyes	371.77	0.99
5	Human	1:2	Medium eyes	309.65	0.96
6	Human	1:2	Small eyes	207.66	0.53
7	Human	1:2.5	Large eyes	164.22	0.17
8	Human	1:2.5	Medium eyes	138.95	0.07
9	Human	1:2.5	Small eyes	106.63	0.02
10	Human	1:3	Large eyes	59.68	0.00
11	Human	1:3	Medium eyes	100.89	0.02
12	Human	1:3	Small eyes	55.25	0.00
13	Animal	1:1.5	Large eyes	284.90	0.92
14	Animal	1:1.5	Medium eyes	377.54	0.99
15	Animal	1:1.5	Small eyes	339.86	0.98
16	Animal	1:2	Large eyes	403.12	1.00
17	Animal	1:2	Medium eyes	249.14	0.79
18	Animal	1:2	Small eyes	303.23	0.95
19	Animal	1:2.5	Large eyes	153.87	0.12
20	Animal	1:2.5	Medium eyes	187.60	0.35
21	Animal	1:2.5	Small eyes	119.23	0.03
22	Animal	1:3	Large eyes	133.35	0.06
23	Animal	1:3	Medium eyes	89.40	0.01
24	Animal	1:3	Small eyes	68.19	0.00
25	Robot	1:1.5	Large eyes	332.09	0.98
26	Robot	1:1.5	Medium eyes	392.34	1.00
27	Robot	1:1.5	Small eyes	298.11	0.94
28	Robot	1:2	Large eyes	261.56	0.85
29	Robot	1:2	Medium eyes	233.36	0.71
30	Robot	1:2	Small eyes	365.19	0.99
31	Robot	1:2.5	Large eyes	177.45	0.26
32	Robot	1:2.5	Medium eyes	129.86	0.05
33	Robot	1:2.5	Small eyes	199.07	0.46
34	Robot	1:3	Large eyes	125.01	0.04
35	Robot	1:3	Medium eyes	76.52	0.01
36	Robot	1:3	Small eyes	149.81	0.10

As described in the calibration procedure, the four head-to-body ratio levels were recoded into a compact-ratio condition for fsQCA. Ratios of 1:1.5 and 1:2 represented membership in the compact-ratio set because they indicated relatively larger heads and more compact body proportions, whereas 1:2.5 and 1:3 represented non-membership because they indicated more elongated body proportions.

#### 3.5.1 Necessity analysis

A necessity analysis was first conducted to test whether any single design element was necessary for high visual attention. As shown in Table 12, no condition—neither the presence nor the absence of any feature—reached the conventional consistency threshold of 0.90. The highest consistency was 0.846 for the compact head-to-body ratio condition. This result indicates that no single design feature constitutes a necessary condition for high visual attention. Therefore, the compact-ratio condition should be interpreted as a recurrent condition in sufficient configurations rather than as a necessary condition.

**Table 12:** Necessity analysis.

Variable	Consistency	Coverage
Human	0.294	0.438
Non-human	0.706	0.525
Animal	0.348	0.517
Non-animal	0.652	0.485
Robot	0.358	0.533
Non-robot	0.642	0.477
Compact head-to-body ratio condition, 1:1.5 or 1:2	0.846	0.839
Elongated head-to-body ratio condition, 1:2.5 or 1:3	0.266	0.263
Large or medium eyes	0.551	0.546
Small eyes	0.510	0.506

#### 3.5.2 Sufficiency analysis

A sufficiency analysis was then performed to identify combinations of conditions sufficient for producing high visual attention. Table 13 presents the three equifinal configurations derived from this analysis. The overall solution consistency was 0.870, exceeding the acceptable threshold of 0.80, and the solution coverage was 0.748, indicating that the three configurations covered a substantial proportion of the high-TFD outcome.

**Table 13:** Sufficient configurations associated with high total fixation duration.

Variable	Configurations		
	1	2	3
Human	×	×	•
Animal	•	×	×
Robot	×	•	×
Compact head-to-body ratio condition, 1:1.5 or 1:2	•	•	•
Large or medium eyes	—	—	•
Raw coverage	0.293	0.291	0.163
Unique coverage	0.293	0.291	0.163
Consistency	0.872	0.867	0.871
Solution coverage	0.748		
Solution consistency	0.870		

‘•’ indicates the presence of a core condition; ‘×’ indicates the absence of a condition; ‘—’ indicates a “don't-care” condition, meaning that the condition was not decisive in the configuration. Core conditions are identified within sufficient configurations and should not be interpreted as necessary conditions unless they meet the necessity threshold.

A key finding across all three configurations was that the compact head-to-body ratio condition (1:1.5 or 1:2) emerged as a core condition shared by all sufficient pathways. Its consistent presence suggests that a head-to-body ratio approximating the infant schema is a highly central design feature for capturing visual attention, even though the necessity analysis confirmed it is not a necessary condition in the strict fsQCA sense. The role of other features, particularly eye size, was contingent on mascot type, as detailed below.

Configuration 1. For Animal mascots, the combination of Animal mascot type and the compact head-to-body ratio condition was sufficient for high visual attention, regardless of eye size (raw coverage = 0.293, consistency = 0.872).

Configuration 2. Similarly, for Robot mascots, the combination of Robot mascot type and the compact head-to-body ratio condition was sufficient for high visual attention, irrespective of eye size (raw coverage = 0.291, consistency = 0.867).

Configuration 3. For Human mascots, large or medium eyes appeared as an additional core condition in the configuration associated with high visual attention.

In summary, the fsQCA results demonstrate that high visual attention arises from multiple equifinal configurations rather than any single optimal design feature. The compact head-to-body ratio condition appeared as the shared core condition across all three pathways, while the role of additional features, particularly large or medium eyes, depended on mascot type. These patterns illustrate the configurational interplay among design elements in guiding visual attention.

## 4 Discussion

This study employed eye-tracking experiments and a subjective preference rating study to examine how mascot type, head-to-body ratio, and eye size shape fixation-based visual attention and self-reported preference among young adults. The eye-tracking results showed that mascot type, head-to-body ratio, and eye-related features were associated with fixation-based visual attention, while the questionnaire results showed a broadly convergent directional pattern in subjective preference ratings. Together, these findings suggest that the visual features associated with stronger fixation-based attention were also evaluated more favorably by participants.

### 4.1 Feature effects and type-contingent patterns

Across the three eye-tracking experiments, mascot type, head-to-body ratio, and eye size each significantly influenced fixation-based visual attention. The strongest descriptive attention patterns were observed for Animal mascots, the 1:2 head-to-body ratio, and large eyes. The subjective preference rating study showed the same directional pattern: these three conditions also received the highest preference scores within their respective design variables. This convergence suggests that the design features associated with stronger visual attention were also more positively evaluated by participants, while fixation duration and subjective preference remain distinct measures.

For mascot type, Animal mascots received the longest fixation duration and the highest preference rating, followed by Robot and Human mascots. This pattern may be related to the ability of animal mascots to combine non-human novelty with recognizable face-like cues (Waytz et al., 2014). Animal forms often allow designers to exaggerate facial expression and body features while maintaining a friendly and approachable character image (Miesler et al., 2011). Therefore, Animal mascots may have an advantage in both attentional engagement and subjective evaluation within the present stimulus set.

For head-to-body ratio, the 1:2 condition received the longest fixation duration and the highest preference score. This result is consistent with baby schema theory, which emphasizes the visual salience of relatively large heads and compact body proportions. However, the fact that 1:2 outperformed 1:1.5 suggests that the most effective proportion is not necessarily the most exaggerated one. Instead, the 1:2 ratio may offer a balance between infantile proportion, visual clarity, and overall acceptability. Baby-schema-like body proportion may enhance attention and preference up to an optimal level, but excessive compression of the body may reduce visual clarity, perceived naturalness, or design acceptability. Thus, in mascot design, infantile proportion may operate as an optimal-intensity cue rather than as a simple “the more exaggerated, the better” principle.

For eye size, mascots with large eyes received the longest fixation duration and the highest preference rating. This finding supports the central role of the eye region in face perception and face-like perception. In mascot design, prominent eyes can function as cues for expression, agency, and social responsiveness, thereby increasing both fixation-based attention and favorable evaluation (Cossu et al., 2024). However, this effect should be understood as the influence of overall eye-related visual design rather than eye size alone.

In the present stimulus set, the eye-size manipulation did not vary only in geometric size. Compared with smaller-eye stimuli, some medium- and large-eye stimuli also showed clearer eye contours, more visible pupil or iris regions, stronger eye–face contrast, and richer ocular expressiveness. These differences may affect fixation allocation and social evaluation, as prior studies have shown that ocular cues such as pupil size, iris brightness, scleral brightness, limbal rings, and iris-related features can influence perceived friendliness, juvenility, health, or attractiveness (Konno et al., 2023; Brown, 2010; Howard, 2026; Wacewicz et al., 2022). Therefore, the present eye-size findings should be interpreted as reflecting the overall ocular-feature configuration rather than eye size alone.

Overall, these feature-effect findings suggest that mascot design features influence both attention-based and self-report measures. However, the effects should not be interpreted as isolated design rules. As shown by the fsQCA results, visual attention to mascots is also shaped by how mascot type, body proportion, and eye-related features are combined.

### 4.2 Theoretical integration and configurational contribution

This study contributes to mascot design research by combining eye-tracking, subjective preference ratings, and fsQCA to examine how mascot design features guide visual attention and preference. Baby schema theory and face-like perception provide interpretive frameworks for discussing the observed patterns. The eye-tracking results showed that mascot type, head-to-body ratio, and eye size each influenced fixation-based visual attention under goal-directed viewing conditions. The subjective preference rating study further showed that the same design features were associated with higher self-reported preference ratings. This convergence suggests that the visual features guiding attention were also positively evaluated by participants, while fixation duration and subjective preference should still be understood as distinct measures.

Some findings can be interpreted in relation to baby schema theory and face-like perception. For head-to-body ratio, the relatively strong performance of the 1:2 condition is broadly consistent with baby schema theory, which emphasizes the salience of relatively large heads and compact body proportions. Baby schema research indicates that infant-like features, such as relatively large heads, enlarged eyes, round faces, and soft contours, can elicit cuteness perception, gaze allocation, and preference (Lorenz, 1943; Glocker et al., 2009; Borgi et al., 2014; Kanwisher et al., 1997). However, the finding that the 1:2 condition outperformed the more exaggerated 1:1.5 condition suggests that the effect of infantile body proportion in mascot design may not follow a simple linear pattern. In the present stimulus set, the 1:1.5 condition may have made the body appear overly compressed, whereas the 1:2 condition may have retained a compact, infant-like proportion while preserving clearer overall form and visual acceptability. Therefore, the advantage of the 1:2 condition should be interpreted cautiously as a finding specific to the present stimulus set, rather than as evidence for a universal optimal head-to-body ratio. Similarly, the results for eye size highlight the importance of the eye region in mascot perception. Prominent eye design may enhance perceived expressiveness, agency, and social responsiveness, thereby contributing to both fixation-based attention and subjective preference.

Beyond these feature-level patterns, the fsQCA results provide a configurational perspective on mascot design. High total fixation duration was associated with multiple sufficient configurations rather than with a single isolated feature. Across the identified configurations, a compact head-to-body ratio condition appeared as a shared core condition, suggesting that infantile body proportions function as an important configurational element in attention-oriented mascot design. The role of eye-related features was more context-dependent: for Animal and Robot mascots, high TFD was associated with a compact head-to-body ratio condition regardless of eye-size condition, whereas for Human mascots, the configuration associated with high TFD included both a compact head-to-body ratio condition and large or medium eyes.

This pattern indicates that mascot design features do not operate in a purely additive way. Instead, the contribution of one feature depends on the broader configuration in which it appears. A feature that is central in one mascot type may be less decisive in another. Therefore, the theoretical contribution of this study lies not only in identifying individual attention-relevant features, but also in showing how mascot type, body proportion, and eye-related features jointly shape visual attention and subjective preference.

### 4.3 Practical implications for design and marketing

The findings offer several implications for mascot design and brand communication. First, Animal mascots showed the strongest fixation-based visual attention and the highest subjective preference ratings among the three mascot types. This suggests that animal forms may be effective when a brand aims to create a visually engaging and favorably evaluated mascot. However, this does not mean that Animal mascots are universally optimal. Brand personality, cultural fit, product category, and desired emotional tone should still guide mascot selection.

Second, compact body proportions, especially proportions close to the 1:2 condition in the present stimulus set, may serve as a useful starting point for attention-oriented and preference-oriented mascot design. For practitioners, a compact body proportion with a relatively large head may serve as a valuable starting point, especially when the design goal is to create an approachable and visually engaging mascot.

Third, eye design should be adjusted according to mascot type. Large or medium eyes may be particularly relevant for Human mascots, whereas Animal and Robot mascots may rely more strongly on body proportion and category-level features. This supports the practical importance of eye-related features in mascot design. However, designers should consider not only eye size but also the broader ocular configuration, including iris brightness, scleral visibility, contrast, and expression. Eye design should be adjusted according to mascot type rather than applied uniformly across all mascot categories.

Finally, the fsQCA results suggest that designers should evaluate combinations of features rather than relying on isolated design rules. A feature that contributes to visual attention in one mascot type may be less decisive in another. Therefore, mascot design should be treated as a configurational process involving type, proportion, and facial-feature arrangement.

### 4.4 Limitations and future research

Several limitations should be noted. First, fixation-based eye-tracking metrics provide useful evidence of visual-attentional allocation, but they do not directly measure emotional response, brand recall, purchase intention, or actual consumer behavior. Although this study added a subjective preference rating task, future research could further incorporate emotional evaluation, memory measures, choice behavior, or purchase-intention indicators to examine how attention and preference translate into consumer outcomes.

Second, the viewing paradigm was goal-directed. Participants were explicitly instructed to attend to specific design features, which helped isolate attention to mascot type, head-to-body ratio, and eye size during deliberate evaluation. However, this paradigm does not capture spontaneous bottom-up attention in natural viewing contexts. In addition, the 30-s exposure duration was selected based on the pilot study to ensure that participants had sufficient time to inspect all stimuli within each task, but it is longer than many real-world advertising or interface-viewing situations. Future studies could compare goal-directed viewing with free-viewing tasks and shorter, more ecologically representative exposure durations to examine whether the same design features guide attention under more naturalistic conditions. Stimulus positions in Experiments 1–3, and the block order in Experiment 4, were fixed across participants to maintain a consistent presentation structure. This may have introduced position- or order-related viewing biases. Future studies should randomize or counterbalance stimulus locations and block order.

Third, although the stimulus sets were designed to control the main manipulated variables within each experiment, they were not fully parametrically controlled across all low-level visual properties. Mascot images may differ in shape, contour, style, emotional expression, and local visual complexity, which may have contributed to the observed fixation patterns. This issue is particularly relevant to the eye-size manipulation. In the present stimulus set, eye size was not isolated as a purely geometric variable. Smaller-eye stimuli often reduced the visible internal structure of the eye, whereas some medium- and large-eye stimuli included more visible pupil or iris regions, clearer eye contours, sclera-like or peri-iridal regions, and stronger eye–face contrast. These uncontrolled ocular differences may have contributed to the observed fixation patterns. Therefore, the eye-size findings should be interpreted as reflecting the overall ocular-feature configuration rather than eye size as an isolated geometric variable.

Fourth, the sample consisted of young Chinese adults aged 18–35. This demographic is relevant to contemporary brand communication, but it does not represent all consumer groups. Future studies should test whether the same attentional and preference patterns appear across different age groups, cultural backgrounds, and consumer segments.

Overall, the study shows that mascot design features influence fixation-based visual attention both independently and in combination, and that the same features also show consistent patterns in subjective preference ratings. Future research can build on this framework by integrating more controlled stimuli, more naturalistic viewing tasks, and broader behavioral outcome measures.

## 5 Conclusions

This study examined how mascot type, head-to-body ratio, and eye-related features are associated with fixation-based visual attention and subjective preference among young adults, using eye-tracking experiments, a questionnaire-based preference rating study, and fsQCA.

The results showed that Animal mascots, the 1:2 head-to-body ratio, and large-eye conditions showed the strongest descriptive attention and preference patterns within their respective design variables. The analyses also indicate that some feature effects, especially head-to-body ratio and eye-related features, should be interpreted in relation to mascot type rather than as fully isolated design rules. The preference rating study provided complementary evidence that attention-related patterns were broadly consistent with self-reported liking.

The fsQCA results further showed that high total fixation duration was associated with multiple sufficient configurations. A compact head-to-body ratio condition, operationalized as 1:1.5 or 1:2, appeared as a shared core condition across the sufficient pathways, but it did not meet the threshold for a necessary condition. Large or medium eyes appeared in the Human mascot pathway, whereas Animal and Robot pathways were associated with compact body proportions regardless of eye-size condition.

These findings support a configurational understanding of mascot design. Visual attention and preference are shaped not only by individual design features, but also by how mascot type, body proportion, and eye-related features are combined. For design practice, compact body proportions and type-appropriate eye design may provide useful starting points, but final design decisions should consider brand positioning, cultural context, and target-user characteristics.

## Data Availability

The original contributions presented in the study are included in the article/supplementary material, further inquiries can be directed to the corresponding author.
